# Incorporating digital anorectal examinations for anal cancer screening into routine HIV care for men who have sex with men living with HIV: a prospective cohort study

**DOI:** 10.1002/jia2.25192

**Published:** 2018-12-05

**Authors:** Jason J Ong, Sandra Walker, Andrew Grulich, Jennifer Hoy, Tim RH Read, Catriona Bradshaw, Marcus Chen, Suzanne M Garland, Richard Hillman, David J Templeton, Jane Hocking, Beng Eu, Bian Kiem Tee, Eric P F Chow, Christopher K Fairley

**Affiliations:** ^1^ Central Clinical School Monash University Melbourne Victoria Australia; ^2^ London School of Hygiene and Tropical Medicine London UK; ^3^ Melbourne Sexual Health Centre Alfred Health Carlton Victoria Australia; ^4^ Kirby Institute University of New South Wales Sydney New South Wales Australia; ^5^ Department of Infectious Diseases Alfred Hospital and Monash University Melbourne Victoria Australia; ^6^ Department of Obstetrics and Gynaecology Department of Microbiology in Infectious Diseases University of Melbourne Royal Women's Hospital Murdoch Children's Research Institute Parkville Victoria Australia; ^7^ HIV, Immunology and Infectious Disease St Vincent's Hospital Darlinghurst New South Wales Australia; ^8^ RPA Sexual Health Sydney Local Health District Sydney Medical School The University of Sydney Sydney New South Wales Australia; ^9^ Melbourne School of Population and Global Health University of Melbourne Parkville Victoria Australia; ^10^ Prahran Market Clinic Prahran Victoria Australia; ^11^ The Centre Clinic Victorian AIDS Council St Kilda Victoria Australia

**Keywords:** anal cancer, cohort studies, coinfection, HIV, key and vulnerable populations, malignancy, men who have sex with men, public health

## Abstract

**Introduction:**

Men who have sex with men (MSM) living with HIV have a high risk of anal cancer, which is often detected at late stages, when morbidity and mortality are high. The objective of this study was to describe the feasibility and challenges to incorporating regular digital anorectal examination (DARE) into routine HIV care for MSM living with HIV, from the perspective of patients, physicians and the health service.

**Methods:**

In 2014, we recruited 327 MSM living with HIV, aged 35 and above from one major sexual health centre (n = 187), two high HIV caseload general practices (n = 118) and one tertiary hospital (n = 22) in Melbourne, Australia. Men were followed up for two years and DARE was recommended at baseline, year 1 and year 2. Data were collected regarding patient and physician experience, and health service use. An ordered logit model was used to assess the relationship between sociodemographic factors and the number of DAREs performed.

**Results:**

Mean age of men was 51 (SD ± 9) years, 69% were Australian born, 32% current smokers, and mean CD4 was 630 (SD ± 265) cells per mm^3^, with no significant differences between clinical sites. Overall, 232 (71%) men received all three DAREs, 71 (22%) received two DAREs, and 24 (7%) had one DARE. Adverse outcomes were rarely reported: anal pain (1.2% of total DAREs), bleeding (0.8%) and not feeling in control of their body during the examination (1.6%). Of 862 DAREs performed, 33 (3.8%) examinations resulted in a referral to a colorectal surgeon. One Stage 1 anal cancer was detected.

**Conclusion:**

Incorporation of an early anal cancer detection programme into routine HIV clinical care for MSM living with HIV showed high patient acceptability, uncommon adverse outcomes and specialist referral patterns similar to other cancer screening programmes.

## 1 Introduction

Men who have sex with men (MSM) living with HIV have a disproportionately high incidence of anal cancer (45.9 per 100,000) compared with HIV‐negative MSM (5.1 per 100,000) 1 making it the most common non‐AIDS defining malignancy in this group 2. The majority of anal cancers are detected late (Stage 2 or above) with associated higher morbidity and mortality, compared with cancers detected at Stage 1 3. If anal cancers can be detected earlier through incorporating regular digital anorectal examination (DARE) into routine clinical care, they may be small enough to excise (if perianal) or require lower doses of chemoradiation with higher survival rates 4. For cancers with a diameter of 2 cm or less, the five‐year disease‐specific survival rate was 80% 5, 6. For cancers <1 cm, one study reported 100% five‐year disease‐specific survival 7.

Currently, the effectiveness of DARE as a screening tool for anal cancer is unknown, but there is mounting evidence to support the utility of DARE. A recent study in men taught self‐examination with DARE found that 3 mm lesions could be detected with a sensitivity of 71% to 80% and specificity of 92% to 100% 8. A centre with over 20 years of experience in providing anal cancer screening with DARE, anal cytology, high‐resolution anoscopy (HRA) and biopsies for MSM living with HIV, presented data on 27 anal cancers detected as part of the screening process 9. Of note, 23 of 27 (85%) had a mass, area of induration or ulcer that could be palpated or seen, detected with an anal examination. Only four men did not have any palpable abnormalities and their cancers detected were solely by vascular changes visualized and biopsied during HRA. Similarly, in another study reporting on 11 anal cancers, 6 of 11 anal cancers were detectable with DARE, 8 of 11 were detectable on clinical inspection (with the naked eye) of the perianal area and external part of the anal canal (after spreading the buttocks). Furthermore, all 11 cancers were detectable using DARE or clinical inspection 10. Together, these studies suggest that sensitivity for anal cancer should be relatively high using DARE and perianal examination as a screening methodology. The specificity of DARE for anal cancer is more difficult to estimate and likely to be lower due to the high prevalence of anorectal pathology in MSM living with HIV and lack of published data regarding whether the screening clinician was concerned about anal cancer or another anorectal pathology 9, 11. Although the majority of final diagnoses from an abnormal DARE will not be anal cancer (i.e. classified as a false positive) 11, a colorectal specialist referral may still be appropriate for management of non‐malignant anorectal pathologies.

To date, only a few international guidelines recommend regular DARE as a screening method for anal cancer 12. From 2016, the Australian HIV Guidelines also recommended an annual DARE for MSM living with HIV age 50 years and above 13. Prior to this, MSM living with HIV in Australia had not had a routine DARE conducted for anal cancer screening and no physicians offered regular DARE as part of their HIV care 14. A recent Australian case series of anal cancer at a Melbourne hospital reported that the mean tumour size was 2.9 cm at diagnosis for people living with HIV 3.

The baseline findings of a study conducted by the authors (ACE: Anal Cancer Examination study) found annual DAREs to be acceptable to the majority of men, with uncommon adverse outcomes such as pain or bleeding after examination 11. We did not find any increase in cancer worry or decrease in quality of life measures at baseline 11. In this paper, we present the follow‐up data from incorporating an annual DARE for two years in a variety of clinical settings. We assessed this from the patient (experience of receiving DARE), physician (experience of conducting DARE, pathological diagnoses identified from DARE) and health services perspectives (colorectal specialist referrals resulting from DARE).

## 2 Methods

### 2.1 Study population

The ACE study was a two‐year prospective cohort study of 327 men, with follow up completed in 2016. Details of the study methodology and recruitment strategy have been published elsewhere 11. Inclusion criteria for patients were MSM living with HIV, who were age 35 years and over, not diagnosed with anal cancer and currently living in Victoria, Australia. Participating physicians were registered medical practitioners who managed MSM living with HIV, and worked in one of the recruitment sites. In Victoria, there is no anal cancer screening programme based on cytology, HPV testing or HRA. Men were recruited by sexual health physicians at one major sexual health centre (n = 187), general practitioners with considerable HIV and sexual health experience at two high HIV case‐load general practices (n = 118, hereafter referred to as “general practice”) and infectious diseases physicians at one tertiary hospital HIV outpatient clinic (n = 22, hereafter referred to as “tertiary hospital”). Written consent was obtained from all participants. To improve the external validity of our study findings, the researchers did not provide extra reminders for men to return for their annual DARE. Each clinic used their own recall systems and prompts in their clinical record to remind men about receiving their DARE when they attended for their clinic appointment.

### 2.2 Measures

Men living with HIV provided written informed consent prior to recruitment to the study. They were recommended to have an annual DARE over two years by their physician (baseline, year 1 and year 2) and asked to complete a questionnaire at each visit. The annual interval was chosen to be in line with men's annual health checks. The physician offered the DARE during the consultation and participants could agree to or decline the examination. Patients were asked about their experience of the DARE including any anal pain, anal bleeding, and whether they felt in control of their body during the examination. HIV‐related information including current CD4 count and HIV viral load was collected from their medical records,. The results of each DARE and the outcomes of any referrals to colorectal surgeons were also obtained by reviewing the clinical records of each participant. At each study time point we reported the number of DAREs categorized as no abnormality detected, diagnoses where the clinician felt there were minor findings (e.g. haemorrhoids) with no need for a referral to a colorectal surgeon and diagnoses that required a referral. The number of DAREs for each clinic, and the proportion of men requiring referral to a colorectal surgeon was calculated per 100 DARE for each study time point and for each clinic. Quality of life was assessed using a generic preference‐based measure of health‐related quality of life, the SF12 (short form health survey) 15, and analysed using the University of Sheffield's SF‐6D scoring method to produce a utility score where one represented perfect health and zero represented a health state equivalent to death 16. The mean utility score at baseline, year 1 and year 2 scores were compared to determine differences in the quality of life.

Training to differentiate potential anal cancer from non‐malignant anorectal pathologies was provided to participating physicians and included practicing a DARE on a model (http://limbsandthings.com/au/products/rectal-examination-trainer-mk-2/), and being shown a website (http://www.anal.org.au/clinician) which contained an educational video that explained the examination, a diagnostic flow chart and an extensive library of photos for common anal conditions. A total of 23 physicians were involved in this study; however three did not return their post‐study questionnaire and were excluded from the analysis. These physicians completed a pre‐ and post‐study questionnaire using a 5‐point Likert scale (very confident to very unconfident) about their confidence conducting DARE, an anoscopy, recognizing anal cancer and managing anal lesions. We collected data about anoscopy as some physicians may use anoscopy for further examining anal canal lesions identified from DARE. We did not provide extra training for anoscopy for physicians in our study. The pre‐study questionnaire was completed prior to a training session for physicians. Physicians were able to access the anal pathologies website as a resource throughout the study. In the post‐study questionnaire they were asked how comfortable they were in recommending DARE to men living with HIV.

### 2.3 Statistical analyses

Descriptive statistics were used to summarize the main outcomes of the study. Pre‐ and post‐study physician questionnaires were analysed for differences with physician confidence and barriers to conducting DARE, over the study period, using McNemar's test. An ordered logit model was used to estimate the relationship between sociodemographic factors from the baseline survey and men who underwent screening. We chose to use this model because our outcome variable had more than two response categories (men who received three DAREs, two DAREs and one DARE). Variables with *p *< 0.2 in univariate analysis were included in the multivariate model. All analyses were performed using STATA version 13.1 (StataCorp LP, College Station, TX, USA).

Our study was reported in line with the STROBE statement for reporting of observational studies 17. This research was approved by the Alfred Hospital Ethics Committee, Melbourne, Australia (Project 246/12).

### 2.4 Role of funding source

The funding body did not have any involvement in the collection, analysis, interpretation of data, writing of the report or the decision to submit for publication.

## 3 Results

The sociodemographic characteristics of the 327 men and recruiting physicians at baseline have been published 11. In brief, the mean age of the participants was 51±9 years and they had lived with HIV for a mean of 13 ± 8 years. Most (69%) were Australian‐born, had completed secondary education or higher (98%), and were virologically suppressed <50 copies/mL (77%), with around one‐third (32%) being current cigarette smokers (32%). Their mean CD4 count was 630 ± 265 cells per mm^3^. There were no statistically significant differences in sociodemographics among participants between the recruiting clinics. Physicians were recruited from a sexual health centre (n = 11), general practice (n = 8) and tertiary hospital (n = 4). The majority were males (61%, n = 14) and aged above 40 years (65%, n = 15).

Of the 327 men recruited at baseline, 268 (82%) received a DARE at their year 1 visit and 267 (81%) at their year 2 visit. In total, 93% of participants had at least one follow‐up DARE during the study while 71% had both follow‐up DAREs. Reasons for not having any DARE after a baseline DARE included: one death, two study withdrawals, four men moved interstate, four men lost to follow up during the study period, four men declined further examinations due to other acute health issues and nine men had no further record of DARE in the clinical notes (unknown whether they were not offered a DARE by their physician or declined one during the clinic consultation). Regarding adverse outcomes, anal pain during the DARE was reported in 10 of 862 DAREs (1.2%, 95% CI: 0.6 to 2.1), anal bleeding in 7 of 862 DAREs (0.8%, 95% CI: 0.3 to 1.7) and not feeling in control of their body during the examination in 14 of the 862 DAREs (1.6%, 95% CI: 0.9 to 2.7).

**Figure 1:**
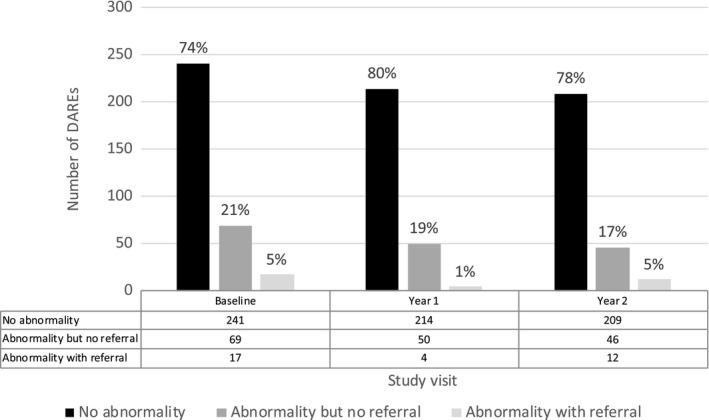
Frequency of abnormalities and referrals from DARE at baseline, year 1 and year 2 visits for men who have sex with men living with HIV (N = 327). DARE, digital anorectal examination.

Of the 862 DAREs performed, 33 (3.8%) examinations in 30 men resulted in a referral to a colorectal surgeon for an anal abnormality detected by the physician performing the DARE. Figure 1 demonstrates the proportions of DARE across the three time points which had no abnormality, an abnormality but no referral and abnormality with referral.

The total proportion of referrals to a colorectal surgeon was 3.8 referrals per 100 DAREs, varying by clinic type (Table 1). The proportion referrals was lowest from the sexual health centre (1.6/100 DARE), followed by general practice (5.4/100 DARE) and the tertiary hospital (16.1/100 DARE).

**Table 1:** DARE diagnoses and proportion referred to a colorectal surgeon for men who have sex with men living with HIV, stratified by recruitment site (N* = *327)

	No abnormality detected	Minor abnormality and no referral	Abnormality and colorectal surgeon referral	Total DARE	Number of referrals to a colorectal surgeon per 100 examinations (95% CI)
Sexual health centre (N = 187)					
Baseline	145	37	5	187	2.7 (0.9 to 6.1)
Year 1	124	31	2	157	1.3 (0.2 to 4.5)
Year 2	136	27	1	164	0.6 (0.0 to 3.4)
Total DARE for Sexual Health	405	95	8	508	1.6 (0.7 to 3.1)
General practice (N = 118)					
Baseline	84	25	9	118	7.6 (3.5 to 14.0)
Year 1	82	13	1	96	1.0 (0.0 to 5.7)
Year 2	65	13	6	84	7.1 (2.7 to 14.9)
Total DARE for General Practice	231	51	16	298	5.4 (3.1 to 8.6)
Tertiary hospital (N = 22)					
Baseline	12	7	3	22	13.6 (2.9 to 34.9)
Year 1	8	6	1	15	6.7 (0.2 to 31.9)
Year 2	8	6	5	19	26.3 (9.1 to 51.2)
Total DARE for Tertiary hospital	28	19	9	56	16.1 (7.6 to 28.3)
All total	664	165	33	862	3.8 (2.6 to 5.3)

95% CI, 95% confidence interval; DARE, digital anorectal examination.

The provisional diagnoses of cases that were referred to a colorectal specialist were: atypical warts (n = 6), deep anal fissures (n = 2), ulceration (n = 5), bleeding lesion in anal canal (n = 1), anal canal lump/polyp (n = 4), anal tenderness that prohibited full DARE (n = 1), PR bleeding with no identifiable cause (n = 1), macular white perianal lesion (n = 1), multiple skin tags (n = 3), prostate lump (n = 2) and no provisional diagnosis (n = 7). The outcomes from the colorectal surgeon referral among 30 participants (three men had two referrals during the study period) were: one man was subsequently diagnosed with anal cancer and treated with chemoradiotherapy, eight men had incidental anal intraepithelial neoplasia (AIN 2‐3) on biopsy of abnormal lesions, eight men with anal warts, skin tag or polyp, two men with an anal fissure, five men who had no anal lesion detected and six men had no record of attendance at a colorectal surgeon appointment.

There were 49 new abnormal anal diagnoses in the follow‐up visits: 27 abnormalities in 268 men at year 1, and 22 abnormalities in 267 men at the year 2 visit. The most common incident diagnoses were skin tags and haemorrhoids followed by anal canal warts (Table 2).

**Table 2:** Number of new abnormalities detected at DARE since the baseline visit for men who have sex with men living with HIV (N = 327)

	Baseline diagnoses	Number of new diagnoses at year 1 (since baseline)	Number;of new diagnoses at year 2 (since baseline and year 1)	Total new diagnoses (since baseline)
Skin tag	21	5	5	10
Haemorrhoids	12	6	4	10
Incidental anal intraepithelial neoplasia 2/3 in biopsy of skin tags	0	3	5	8
Intracanal warts	24	5	2	7
Intracanal polyp/cyst	6	1	3	4
Intracanal lump – diagnosis unclear	6	1	3	4
Anal fissure	3	2	1	3
Perianal ulcer – diagnosis unclear	9	2	1	3
Intracanal thickening – diagnosis unclear	1	2	1	3
Perianal psoriasis	3	2	0	2
Perianal lichenification	2	0	1	1
Perianal tinea	1	0	1	1
Enlarged prostate	9	1	0	1
Perianal infection/abscess	1	0	0	0
Intracanal anal cancer	1	0	0	0
Intracanal scar tissue	1	0	0	0

DARE, digital anorectal examination.

Table 3 summarizes the ordered logistic regression results for factors associated with receiving follow‐up DAREs. In the bivariate analysis, the following factors were associated with having one additional follow‐up DARE (referent category: no follow‐up DAREs): age above 50 years, holding a healthcare card, being an ex‐smoker, and those attending a sexual health centre. In the multivariable analysis, only age 50 years and above, healthcare card holders, and ex‐smokers (relative to men who never smoked) remained statistically significantly associated with having one additional follow‐up DARE.

**Table 3:** Association between number of DAREs completed with sociodemographic factors of men who have sex with men living with HIV, using ordered logistic regression

	n	Unadjusted odds ratios (95% CI)	*p* value	Adjusted odds ratios^c^ (95% CI)	*p* value
Age group^a^					
<50 years	163	1		1	
≥50 years	164	2.47 (1.51 to 4.05)	<0.001	2.59 (1.41 to 4.74)	<0.01
Recruitment site					
General practice	118	1		1	
Sexual health centre	187	1.73 (1.05 to 2.85)	0.03	1.63 (0.91 to 2.92)	0.10
Tertiary hospital	22	1.11 (0.42 to 2.93)	0.83	0.82 (0.27 to 2.48)	0.73
HIV duration (per year increase)	327	1.02 (0.99 to 1.06)	0.16	1.01 (0.97 to 1.04)	0.72
Healthcare card holder^b^					
No	204	1		1	
Yes	108	1.81 (1.05 to 3.14)	0.03	1.96 (1.04 to 3.70)	0.04
Smoking status					
Never smoked	104	1		1	
Ex‐smoker	107	2.33 (1.27 to 4.27)	0.01	2.10 (1.07 to 4.14)	0.03
Current smoker	100	1.55 (0.86 to 2.77)	0.14	1.90 (0.97 to 3.72)	0.06
Number of lifetime partners for receptive anal sex					
0 to 10 men	71	1		1	
11 to 50 men	89	1.26 (0.63 to 2.52)	0.17	1.67 (0.78 to 3.58)	0.19
51 to 100 men	38	1.56 (0.62 to 3.93)	0.13	2.68 (0.89 to 8.05)	0.08
>100 men	100	0.71 (0.38 to 1.35)	0.50	0.78 (0.38 to 1.58)	0.49

a Age group cutoff was chosen as the recommended screening age for an annual DARE in Australia is for men age 50 years and above.

b Healthcare cards are given to Australian residents with a lower income for access to cheaper medicines and other medical care.

c Adjusted for age group, recruitment site, HIV duration, healthcare card holder, smoking status and number of lifetime partners.

Of the 241 men who completed the final questionnaire, 229 (95%, 95% CI: 91 to 97) reported they would continue to have an annual DARE as part of routine screening in the future, 190 (79%, 95% CI: 73 to 84) felt more likely to consult a doctor if they found an abnormality themselves or had anal symptoms, and 217 (90%, 95% CI: 86 to 94) were more comfortable discussing matters regarding anal health with their doctor as a result of their experience in the study. Of the 33 men who were referred for an anal abnormality, 92% (95% CI: 76 to 98) were also likely to continue having an annual DARE. In assessing the impact of DARE on quality of life, we found a mean utility score of 0.75 (standard deviation 0.13) at baseline, 0.76 (standard deviation 0.14) at the year 1 visit and 0.74 (standard deviation 0.13) at the year 2 visit, the change between three visits was not statistically significant (*p* = 0.40).

### 3.1 Physician experience with the ACE study

#### 3.1.1 Overall confidence with DARE over the study period

Physicians reported the following barriers to conducting an annual DARE: lack of consultation time (n = 7, 39%), the patient was not interested (n = 4, 22%), patient declined as they expressed discomfort with DARE (n = 4, 22%), the physician was not comfortable conducting a DARE (n = 1, 6%) and that the physician forgot offer a DARE (n = 2, 11%). These barriers did not differ across clinic types. At the end of the study, all physicians who completed a questionnaire (n = 20, 100%) would recommend DARE to their patients if a DARE‐based screening programme was introduced and included in HIV management guidelines.

Among physicians who completed both questionnaires (Table 4), there were no differences before and after the study in physicians’ experiences with DARE with the majority feeling confident or very confident in performing DARE, performing anoscopy, recognizing an anal cancer and managing an anal lesion (all *p* > 0.3). There was a difference between clinic types, with infectious diseases physicians from the tertiary hospital least likely (compared with physicians from general practices and the sexual health centre) to feel they could recognize an anal cancer or perform an anoscopy at both the beginning and the end of the study (*p *< 0.05).

**Table 4:** Pre‐ and post‐study physician experience with conducting DARE (N = 20)

Overall physician experiences	Pre study n(%)	Post study n(%)	*p* value^a^
Confidence in ability to perform DARE			0.28
Confident	18 (90)	19 (95)	
Unconfident or neither confident nor unconfident	2 (10)	1 (5)	
Confidence in ability to perform an anoscopy			0.97
Confident	15 (75)	15 (75)	
Unconfident or neither confident nor unconfident	5 (25)	5 (25)	
Confidence in ability to recognize an anal cancer			0.32
Confident	15 (75)	17 (85)	
Unconfident or neither confident nor unconfident	5 (25)	3 (15)	
Confidence in managing a lesion that may be anal cancer			0.57
Confident	16 (80)	17 (85)	
Unconfident or neither confident nor unconfident	4 (20)	3 (15)	

a *p* value was calculated from McNemar's test.

## 4 Discussion

Much has been published on cytology‐based anal cancer screening but little is known about the role of DARE, particularly when HRA is not available 18. Unlike previous publications where DARE was examined cross‐sectionally or over a short period 19, 20, we present prospective data over two years from a variety of clinical settings. We assessed this from the patient, physician and health services perspectives to contribute to the scant literature on the incorporation of regular DARE into routine HIV care for MSM living with HIV. Reporting and recall bias from patients were minimized by corroborating outcomes from DARE from their clinical notes. We have demonstrated the incorporation of an early anal cancer detection programme into routine HIV clinical care, in different clinical settings (tertiary hospital, sexual health clinic and general practice) is feasible. We found a high and sustained proportion of patient participation in receiving an annual DARE, with infrequent reports of adverse outcomes. Physicians diagnosed a variety of abnormalities using DARE including the detection of one anal cancer, with the proportion of men requiring colorectal specialist referrals lower than that reported from colorectal cancer screening programmes 21. These findings together with the recent study showing that DARE is cost‐effective 22 provide additional support for its routine implementation into the care of MSM living with HIV.

Incorporating regular DARE into routine HIV care for MSM living with HIV is acceptable to patients, with uncommon reports of adverse outcomes. In our study, the majority of men underwent all three DAREs that were recommended over the period (71%), or two DAREs over the two‐year study period (22%). If DARE is to be scaled up in settings where clinicians and patients may be less motivated, strategies to increase the awareness of anal cancer risk 23 and addressing clinician and clinical factors will be paramount 24. Like other screening programmes 21, 25, 26, repeating the test at regular intervals increases the likelihood of cancer detection. Importantly, we found ancillary benefits from participating in the study: nearly all men reported they would continue receiving an annual DARE post‐study, and the majority were more likely to consult a doctor for future anal abnormalities and felt more comfortable in discussing matters related to anal health with their doctor. These changes in behaviours are particularly salient given the current long lead‐time with a mean of 22 weeks between symptoms of anal cancer and diagnosis by a doctor 3. Greater awareness of anal cancer and greater comfort in discussing anal health issues with a health provider may contribute to earlier anal cancer detection, similar to the experience of testicular cancer screening 27.

The majority of physicians continued to feel comfortable conducting DAREs over the two years of follow up, diagnosing a variety of anal pathologies and referring to a colorectal surgeon. In keeping with an anal cancer incidence of approximately 1 in 2000 men per year in MSM living with HIV 1, the majority of examinations in our study were either normal or detected a benign anorectal lesion. Physicians who incorporate DARE for anal cancer screening, should either be competent in diagnosing and managing the wide variety of anorectal conditions or easily refer common anorectal conditions. Furthermore, to aid physicians in detecting early anal cancers, it would be useful for the establishment of an anal cancer registry that collects standardized data on clinical symptoms and examination findings of anal cancers. To date, there are no data on the clinical features of early anal cancer as the majority present in late stages 3.

Our study provides reassurance at the health service level that the proportion of men needing colorectal specialist referrals remained low over the study period. The proportion of DAREs referred was 4% which is less than the rate of 7% for men undergoing colorectal cancer screening 21. However, there were differences in referral patterns between those practicing in a sexual health centre, general practice or tertiary hospital. This may reflect differences in either the patient cohort and anal pathologies detected and/or the physician's specialty training and experience in managing anorectal conditions. While we provided in‐person training and access to website videos to all participating physicians 19, there remains a learning curve for those physicians for whom anorectal examinations are not part of routine practice. In a centre with over two decades of anal cancer screening experience using DARE, anal cytology and HRA, 85% of anal cancers were detectable using DARE with the remainder detected during high resolution anoscopy 9. Encouragingly, it has been reported that anal canal lesions as small as 3 mm can be detected with a sensitivity of 80% and specificity of 100% 28. As physicians continue to gain experience in DARE, the sensitivity and specificity should improve over time and thus contribute to the screening procedure becoming more cost‐effective 22.

This study should be read in the light of its limitations. First, physician factors (lack of time, forgetfulness) as well as patient factors (competing health priorities during the consultation) meant that not all men received an annual DARE. However, our data reflect the normal course of doctor‐patient interactions and give a pragmatic estimate of the likelihood of men having an annual DARE. Second, the study was confined to inner city clinics in Victoria, Australia and so may not be generalizable to other risk groups such as transplant recipients, rural and remote settings or countries with different healthcare settings or systems. Furthermore, men who participated in the study may have heightened interest in anal cancer and be more motivated to complete their follow‐up DAREs. Third, the majority of participants were from sexual health clinic and general practices where clinicians may be more experienced with DARE and in managing anorectal conditions.

## 5 Conclusion

Incorporation of an annual DARE into HIV care is feasible, if staff members can be appropriately trained. DARE was acceptable to MSM living with HIV and to their physicians. Implementing an annual DARE increased opportunities for discussion on anal health between physicians and patients.

## Competing interests

All authors declare that there are no conflicts of interest.

## Authors’ contributions

JO, CF, AG, JH, MC and SG designed the study. JO, SW, MC, TR, CB, JH, BE and BT contributed to data collection. JO, CF, SW, TR, CB, RH, DT, JH and EC analysed the data and revised the manuscript. All authors read and approved the final manuscript.
